# Supplementary material for: Temperature Evolution of Quasi-one-dimensional C60 Nanostructures on Rippled Graphene
Source: Sci Rep. 2015 Sep 22;5:14336. doi: 10.1038/srep14336 (PMC4585716; doi:10.1038/srep14336)
Supplement: Supplementary Information [file srep14336-s1.pdf]

Supplementary Information

Temperature Evolution of Quasi-one-dimensional C_{60} Nanostructures on Rippled Graphene

Chuanhui Chen, Husong Zheng, Adam Mills, James R. Heflin, and Chenggang Tao*

Department of Physics, Virginia Tech, Blacksburg, Virginia 24061, United States

* Correspondence should be addressed to Chenggang Tao (E-mail: cgtao@vt.edu).

1. Type A and type B facets after 423 K annealing

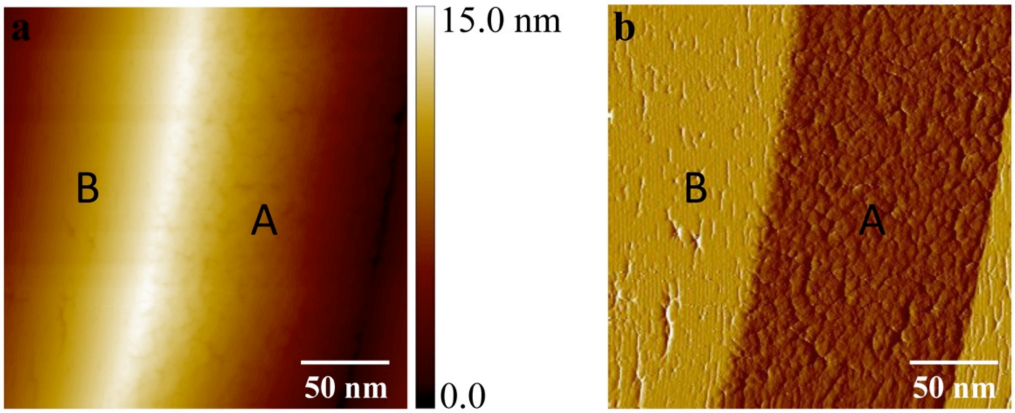

Fig. S1. STM images of type A and type B facets with C_{60} molecules covered after 423 K annealing. (a) The topography and (b) corresponding current images of type A and type B facets with C_{60} after 423 K annealing (V_s = 2.00 V, I = 0.30 nA).

After annealing at a temperature of 423 K, the surface shows two types of facets with well-distinguishable surface features. Fig.S1 (a) shows the large scale STM image of these two types of facets, labeled as A and B. The left side of this image is a facet with the ordered C_{60} chain structure, referred to as type B. The area labeled as A is less-ordered with C_{60} closed packed island structure, referred to as type A. The bottom right corner of the image shows another type B facet, which is parallel to the facet on the left side. Fig.S1 (b) is the corresponding current image, clearly showing the well-ordered C_{60} structures on type B facets and less-ordered C_{60} structures on type A facets. In this large scale image, the well-ordered 1D features can be easily observed.

2. Type A and type B facets after 487 K annealing

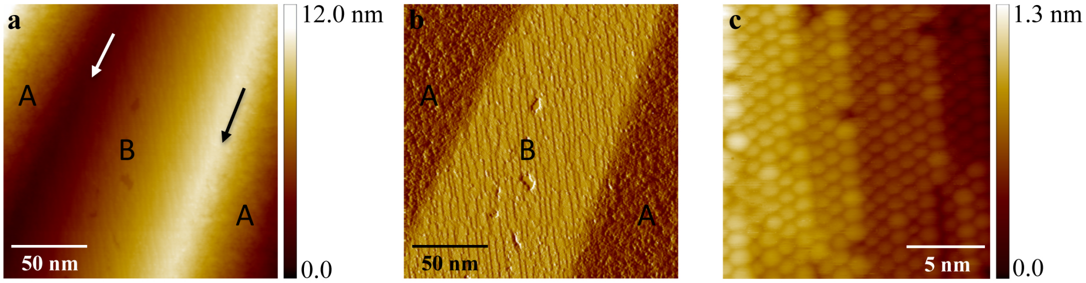

Fig. S2. STM images of type A and type B facets with C_{60} molecules covered after 487 K annealing. (a) The topography and (b) corresponding current images of type A and type B facets with C_{60} after 487 K annealing (V_s = 1.50 V, I = 0.30 nA). (c) High resolution STM image of C_{60} stripes with different widths on type B facets ((V_s = − 2.30 V, I = 0.20 nA).

After annealing at a temperature of 487 K, the surface again shows the typical well ordered and less ordered facets, referred to as type B and A respectively. Fig.S2 (a) shows the large scale STM image with both well-ordered (type B) and less-ordered facets (type A). The left and right sides are type A facets with a type B facet in the middle. Here we can see the connecting lines between vicinal type A and type B facets, which appear as either a protrusion (marked by the black arrow) or a depression line (marked by the white arrow), reflecting the different angles between the vicinal facets. Fig.S2 (b) is the corresponding current image, clearly showing the well-ordered C_{60} quasi-1D structures on type B facets and less-ordered C_{60} structures on type A facets.

3. Close packed C_{60} islands and atomic resolution STM images of flat graphene

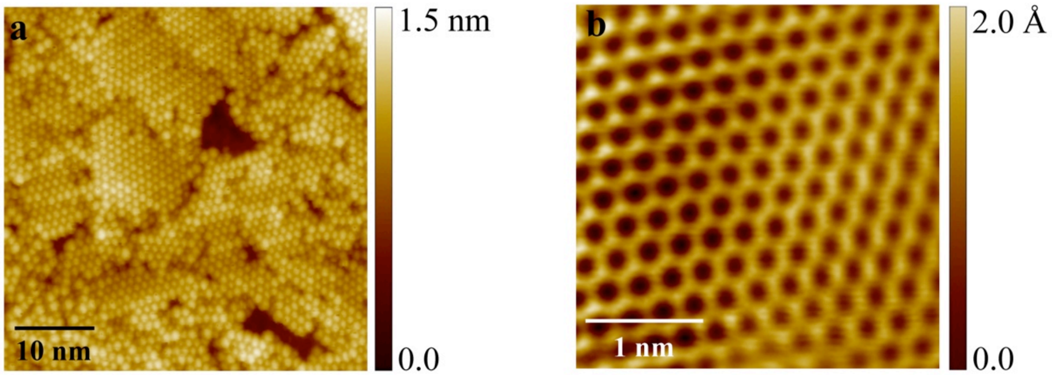

Fig. S3. (a) The high-resolution STM image of C_{60} islands on type A facets (V_s = 1.20 V, I = 0.50 nA). (b) Atomic resolution STM image of flat graphene on an empty area adjacent to a close-packed C_{60} island (V_s = 0.10 V, I = 3.50 nA).

From the large scale STM images in Fig. S1 and Fig.S2, C_{60} in the less-ordered phase is always on type A facets. Fig.S3 (a) is the typical C_{60} structure on a type A facet with high resolution. From it we can clearly see C_{60} close packed small islands with relatively random orientations. Fig. S3 (b) is the atomic resolution STM image of flat graphene on an empty area adjacent to a close-packed C_{60} island.
